# Supplementary figures and images for: Early detection and analysis of accurate breast cancer for improved diagnosis using deep supervised learning for enhanced patient outcomes
Source: PeerJ Comput Sci. 2025 Apr 24;11:e2784. doi: 10.7717/peerj-cs.2784 (PMC12190644; doi:10.7717/peerj-cs.2784)

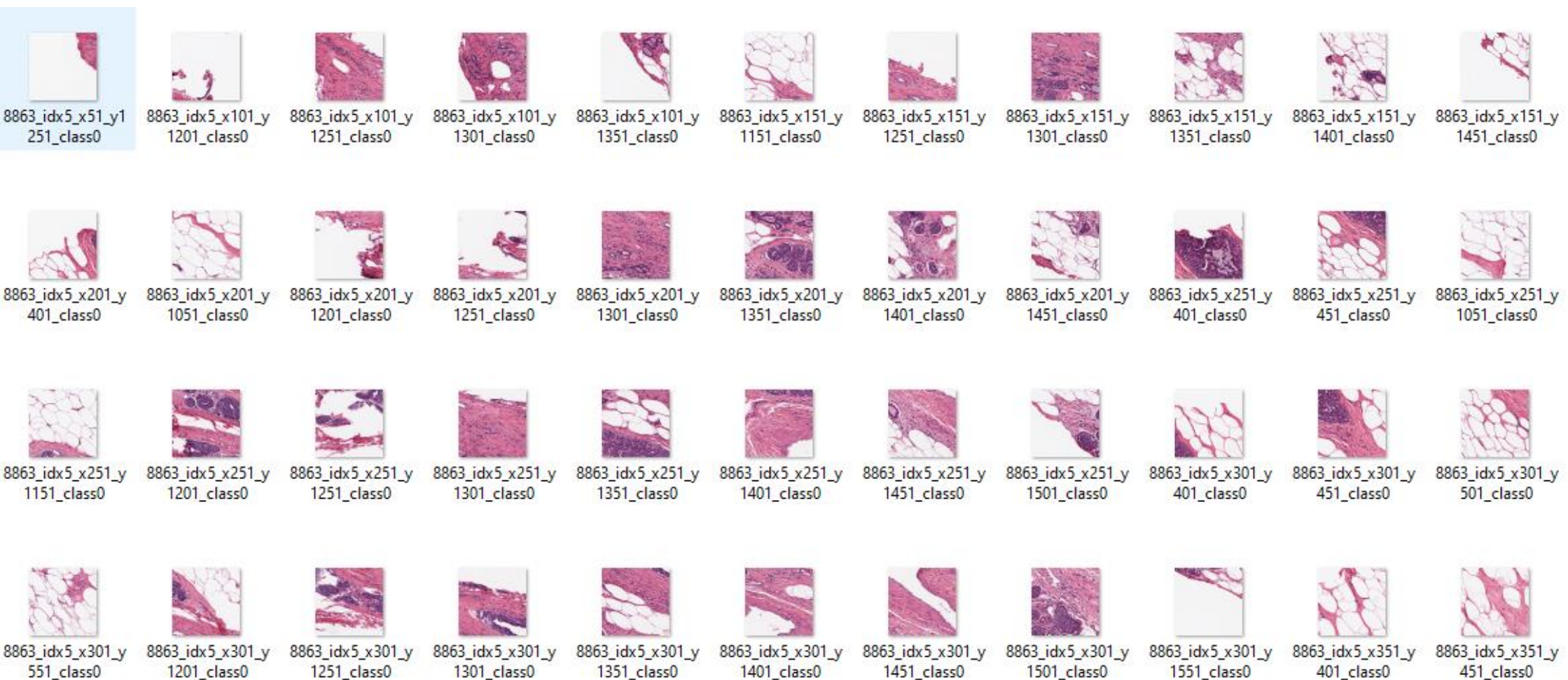

Supplement: Supplemental Information 3 — An overview of the BreaKHis dataset which contains histopathological images of breast cancer including various labeled images categorized by tumor type and grade, which are used for training and evaluating machine learning models for breast cancer detection and classification. [file peerj-cs-11-2784-s003.pdf]

## Correlation of Features

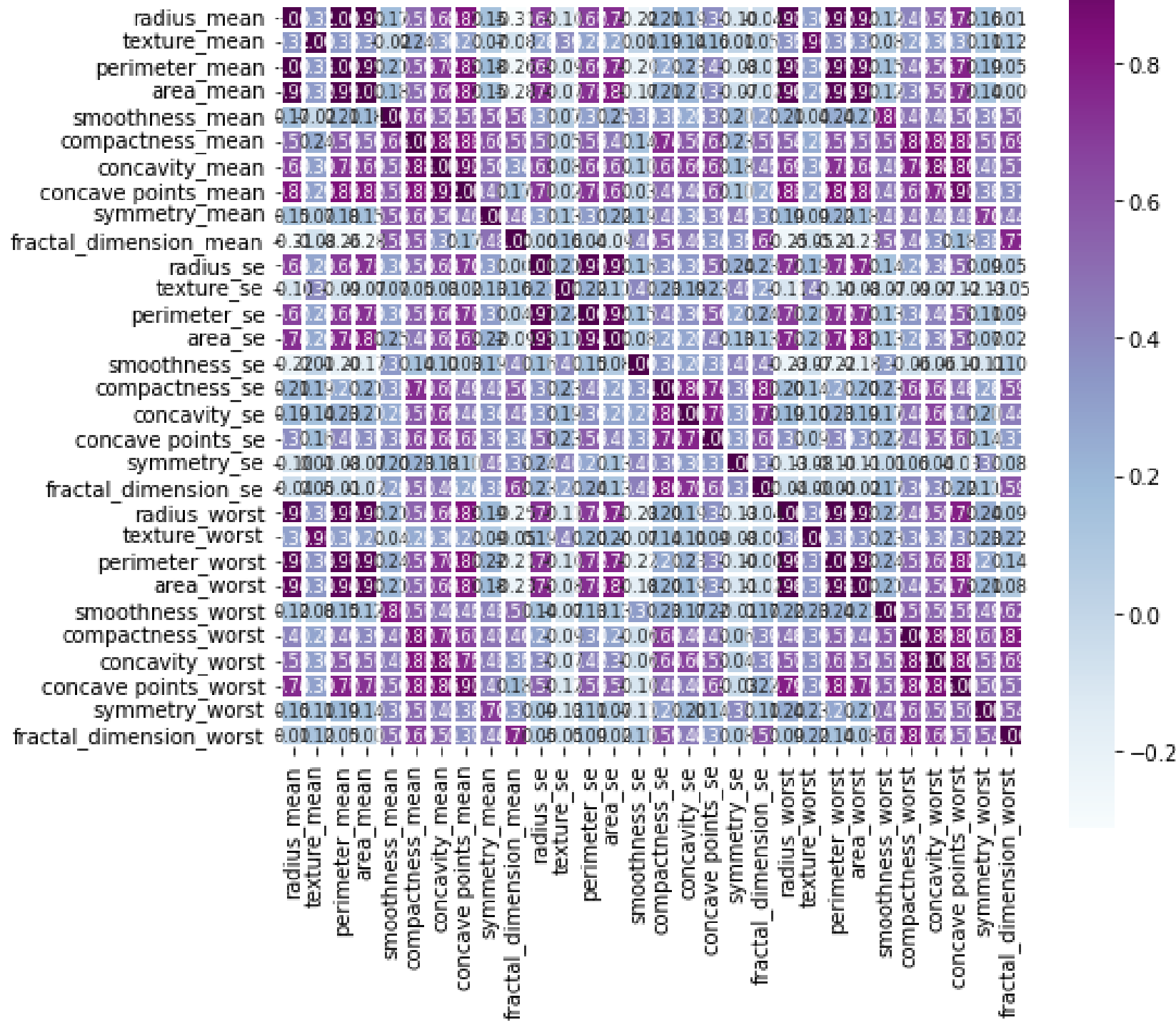

Supplement: Supplemental Information 6 — The correlation between various features in the dataset, providing insights into the relationships between variables. Strong positive or negative correlations are highlighted, helping to identify patterns or redundancies in the data that may influence model performance and feature selection. [file peerj-cs-11-2784-s006.pdf]
