# Supplementary material for: Early detection and analysis of accurate breast cancer for improved diagnosis using deep supervised learning for enhanced patient outcomes
Source: PeerJ Comput Sci. 2025 Apr 24;11:e2784. doi: 10.7717/peerj-cs.2784 (PMC12190644; doi:10.7717/peerj-cs.2784)
Supplement: Supplemental Information 4 — The pre-processing techniques applied to the dataset, including image resizing, normalization, augmentation, and noise reduction. These steps ensure that the dataset is standardized and ready for input into machine learning models, improving the accuracy and robustness of the classification process. [file peerj-cs-11-2784-s004.pdf]

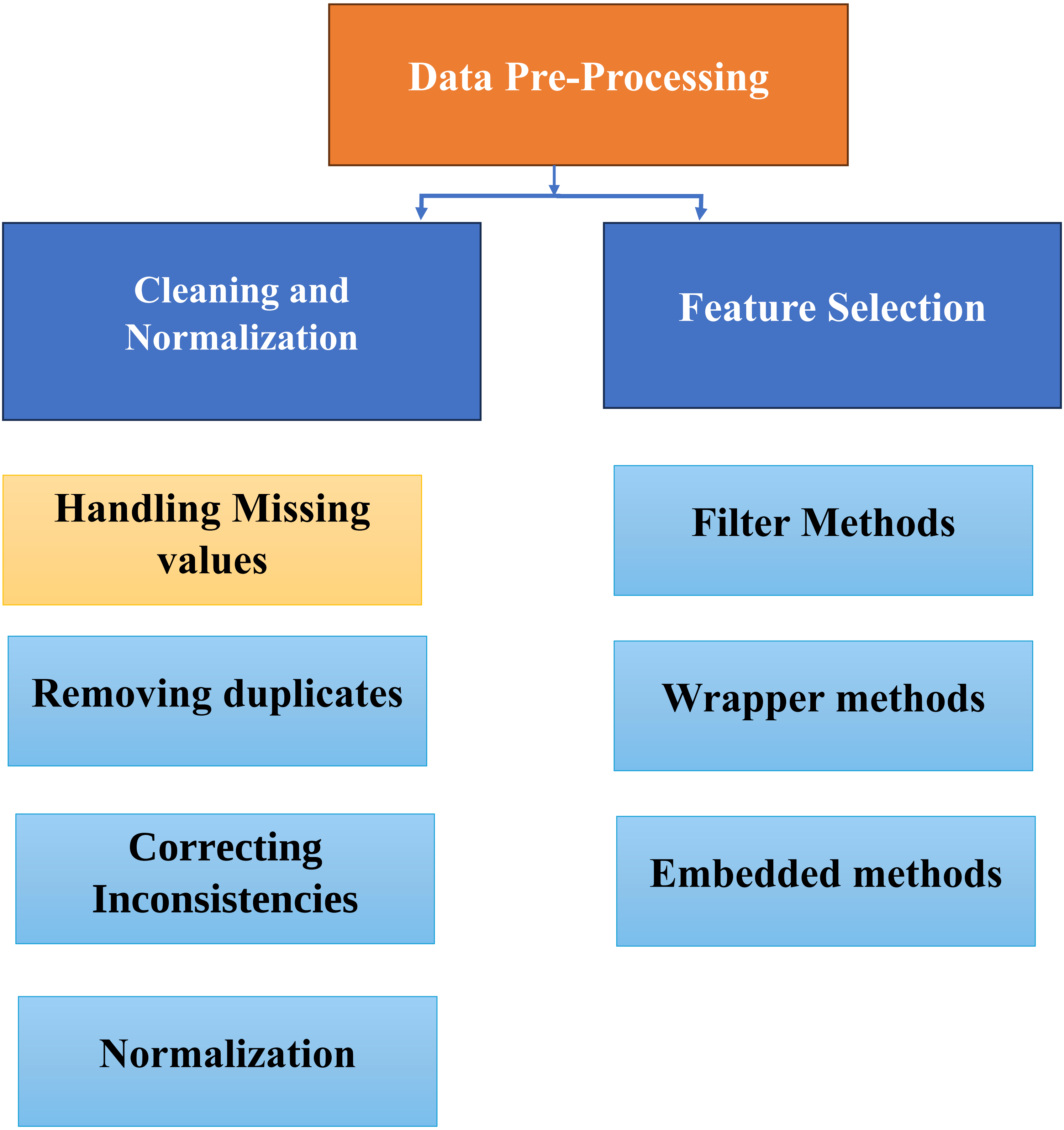
Data Pre-Processing
Cleaning and Normalization
Feature Selection
Handling Missing values
Removing duplicates
Correcting Inconsistencies
Normalization
Filter Methods
Wrapper methods
Embedded methods
