# Supplementary material for: Early detection and analysis of accurate breast cancer for improved diagnosis using deep supervised learning for enhanced patient outcomes
Source: PeerJ Comput Sci. 2025 Apr 24;11:e2784. doi: 10.7717/peerj-cs.2784 (PMC12190644; doi:10.7717/peerj-cs.2784)
Supplement: Supplemental Information 5 — The application of the Local Outlier Factor (LOF) method for detecting outliers in the dataset. (A) the distribution of data points and their corresponding outlier scores, highlighting the potential anomalies. (B) the identified outliers, along with the data points and their respective outlier scores, helping to visualize how the LOF method distinguishes unusual or extreme values in the dataset. [file peerj-cs-11-2784-s005.pdf]

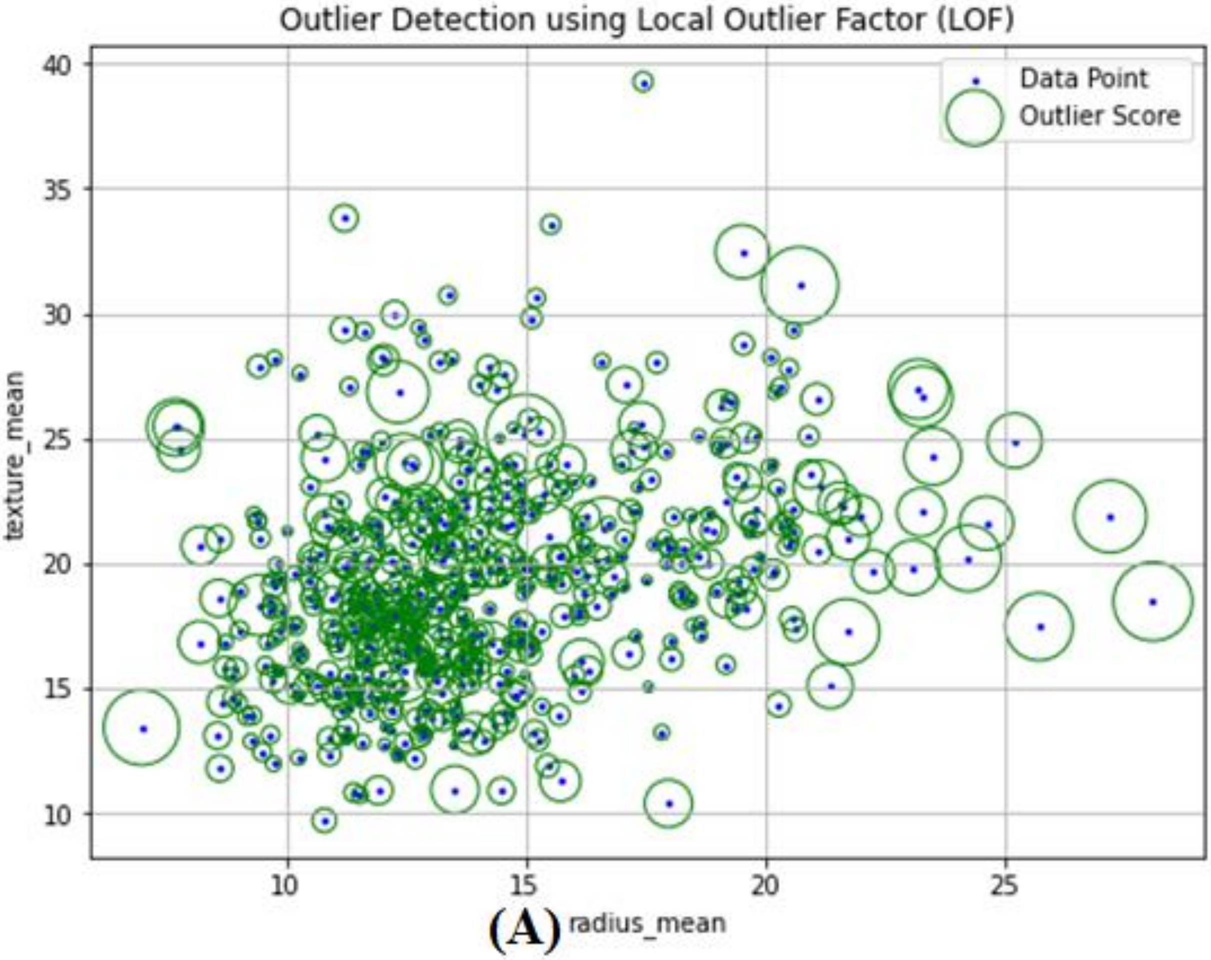
Outlier Detection using Local Outlier Factor (LOF)
Data Point
Outlier Score
texture_mean
radius_mean
40
35
30
25
20
15
10
10
15
20
25
(A)

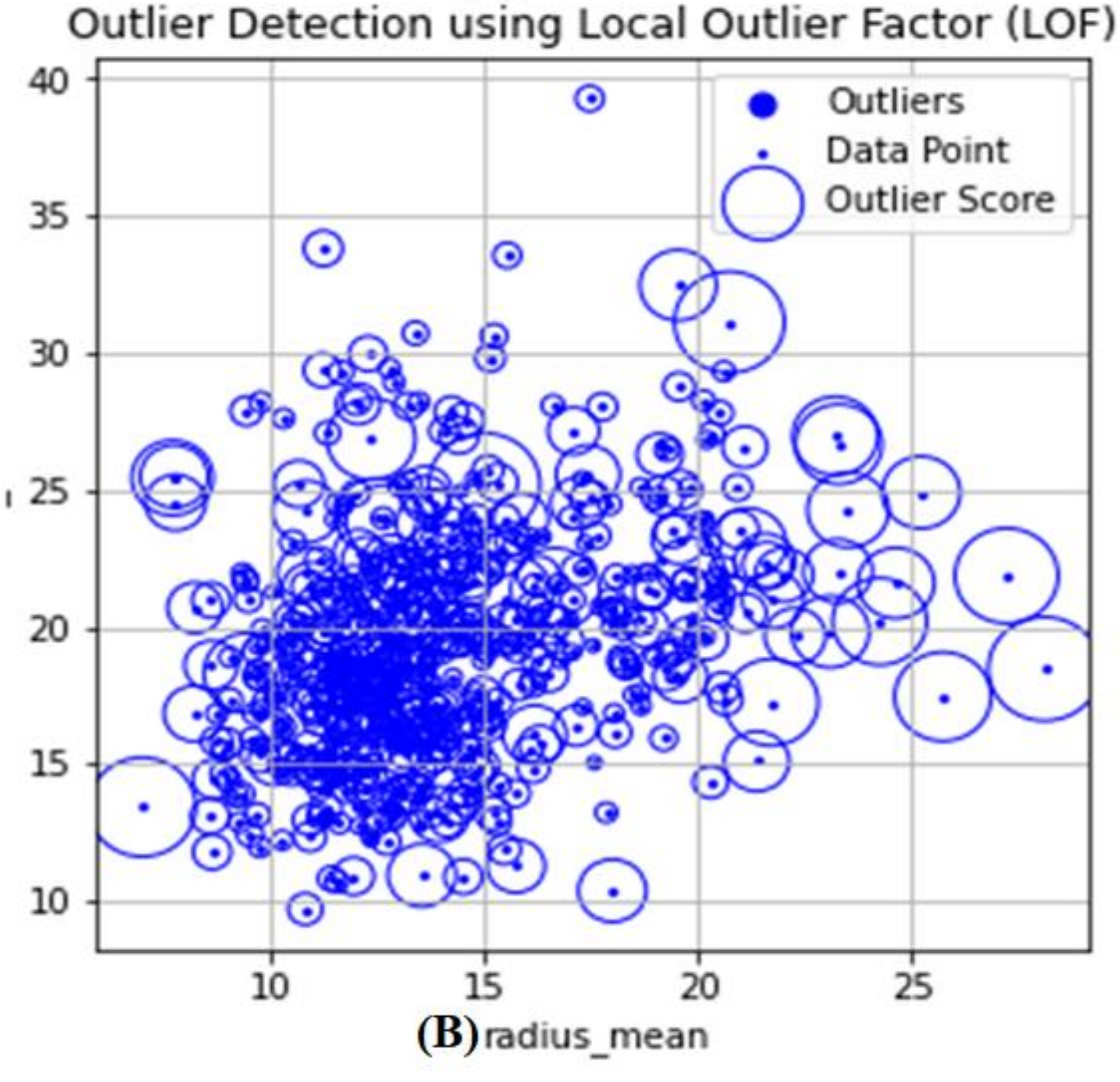
Outlier Detection using Local Outlier Factor (LOF)
Outliers
Data Point
Outlier Score
radius_mean
40
35
30
25
20
15
10
10
15
20
25
(B)
