# Supplementary material for: Early detection and analysis of accurate breast cancer for improved diagnosis using deep supervised learning for enhanced patient outcomes
Source: PeerJ Comput Sci. 2025 Apr 24;11:e2784. doi: 10.7717/peerj-cs.2784 (PMC12190644; doi:10.7717/peerj-cs.2784)
Supplement: Supplemental Information 7 — A detailed summary of the models employed in the research, including their architecture, key parameters, and performance metrics such as accuracy, precision, recall, and F1-score. These metrics provide a comparative overview of how each model performed in the breast cancer detection task. [file peerj-cs-11-2784-s007.docx]

**Supplementary Table 1**: Model Summary

Model: "sequential"

_________________________________________________________________

Layer (type) Output Shape Param #

=================================================================

conv1d (Conv1D) (None, 29, 16) 48

batch_normalization (Batch N (None, 29, 16) 64

ormalization)

dropout (Dropout) (None, 29, 16) 0

conv1d_1 (Conv1D) (None, 28, 32) 1056

batch_normalization_1 (Batc (None, 28, 32) 128

hNormalization)

dropout_1 (Dropout) (None, 28, 32) 0

flatten (Flatten) (None, 896) 0

dense (Dense) (None, 32) 28704

dropout_2 (Dropout) (None, 32) 0

dense_1 (Dense) (None, 1) 33

=================================================================

Total params: 30,033

Trainable params: 29,937

Non-trainable params: 96

Epoch 1/35

16/16 [==============================] - 5s 79ms/step - loss: 0.7427 - accuracy: 0.6250 - val_loss: 0.6199 - val_accuracy: 0.8246

Epoch 2/35

16/16 [==============================] - 0s 26ms/step - loss: 0.4978 - accuracy: 0.7793 - val_loss: 0.5802 - val_accuracy: 0.8596

Epoch 3/35

16/16 [==============================] - 0s 14ms/step - loss: 0.3812 - accuracy: 0.8359 - val_loss: 0.5398 - val_accuracy: 0.8596

Epoch 4/35

16/16 [==============================] - 0s 13ms/step - loss: 0.2881 - accuracy: 0.8867 - val_loss: 0.5021 - val_accuracy: 0.8596

Epoch 5/35

16/16 [==============================] - 0s 11ms/step - loss: 0.2508 - accuracy: 0.9004 - val_loss: 0.4646 - val_accuracy: 0.8772

Epoch 6/35

16/16 [==============================] - 0s 9ms/step - loss: 0.2276 - accuracy: 0.9102 - val_loss: 0.4288 - val_accuracy: 0.8772

Epoch 7/35

16/16 [==============================] - 0s 10ms/step - loss: 0.2294 - accuracy: 0.9277 - val_loss: 0.3956 - val_accuracy: 0.8596

Epoch 8/35

16/16 [==============================] - 0s 26ms/step - loss: 0.1818 - accuracy: 0.9355 - val_loss: 0.3672 - val_accuracy: 0.8421

Epoch 9/35

16/16 [==============================] - 0s 12ms/step - loss: 0.1923 - accuracy: 0.9121 - val_loss: 0.3456 - val_accuracy: 0.8421

Epoch 10/35

16/16 [==============================] - 0s 13ms/step - loss: 0.1671 - accuracy: 0.9395 - val_loss: 0.3256 - val_accuracy: 0.8421

Epoch 11/35

16/16 [==============================] - 0s 14ms/step - loss: 0.1770 - accuracy: 0.9277 - val_loss: 0.3068 - val_accuracy: 0.8421

Epoch 12/35

16/16 [==============================] - 0s 10ms/step - loss: 0.1618 - accuracy: 0.9316 - val_loss: 0.2881 - val_accuracy: 0.8596

Epoch 13/35

16/16 [==============================] - 0s 11ms/step - loss: 0.1413 - accuracy: 0.9512 - val_loss: 0.2743 - val_accuracy: 0.8596

Epoch 14/35

16/16 [==============================] - 0s 10ms/step - loss: 0.1330 - accuracy: 0.9414 - val_loss: 0.2579 - val_accuracy: 0.8596

Epoch 15/35

16/16 [==============================] - 0s 10ms/step - loss: 0.1471 - accuracy: 0.9414 - val_loss: 0.2507 - val_accuracy: 0.8596

Epoch 16/35

16/16 [==============================] - 0s 12ms/step - loss: 0.1148 - accuracy: 0.9688 - val_loss: 0.2333 - val_accuracy: 0.8596

Epoch 17/35

16/16 [==============================] - 0s 11ms/step - loss: 0.1148 - accuracy: 0.9570 - val_loss: 0.2185 - val_accuracy: 0.8772

Epoch 18/35

16/16 [==============================] - 0s 9ms/step - loss: 0.1157 - accuracy: 0.9570 - val_loss: 0.2049 - val_accuracy: 0.9123

Epoch 19/35

16/16 [==============================] - 0s 9ms/step - loss: 0.1180 - accuracy: 0.9609 - val_loss: 0.1942 - val_accuracy: 0.9123

Epoch 20/35

16/16 [==============================] - 0s 11ms/step - loss: 0.1043 - accuracy: 0.9688 - val_loss: 0.1886 - val_accuracy: 0.9123

Epoch 21/35

16/16 [==============================] - 0s 7ms/step - loss: 0.0959 - accuracy: 0.9707 - val_loss: 0.1773 - val_accuracy: 0.9123

Epoch 22/35

16/16 [==============================] - 0s 8ms/step - loss: 0.1144 - accuracy: 0.9551 - val_loss: 0.1659 - val_accuracy: 0.9298

Epoch 23/35

16/16 [==============================] - 0s 8ms/step - loss: 0.1268 - accuracy: 0.9551 - val_loss: 0.1580 - val_accuracy: 0.9298

Epoch 24/35

16/16 [==============================] - 0s 9ms/step - loss: 0.1019 - accuracy: 0.9590 - val_loss: 0.1490 - val_accuracy: 0.9298

Epoch 25/35

16/16 [==============================] - 0s 9ms/step - loss: 0.1045 - accuracy: 0.9629 - val_loss: 0.1402 - val_accuracy: 0.9298

Epoch 26/35

16/16 [==============================] - 0s 9ms/step - loss: 0.0924 - accuracy: 0.9727 - val_loss: 0.1339 - val_accuracy: 0.9298

Epoch 27/35

16/16 [==============================] - 0s 11ms/step - loss: 0.0859 - accuracy: 0.9648 - val_loss: 0.1304 - val_accuracy: 0.9474

Epoch 28/35

16/16 [==============================] - 0s 11ms/step - loss: 0.0913 - accuracy: 0.9727 - val_loss: 0.1238 - val_accuracy: 0.9474

Epoch 29/35

16/16 [==============================] - 0s 12ms/step - loss: 0.0892 - accuracy: 0.9668 - val_loss: 0.1187 - val_accuracy: 0.9649

Epoch 30/35

16/16 [==============================] - 0s 11ms/step - loss: 0.0777 - accuracy: 0.9688 - val_loss: 0.1141 - val_accuracy: 0.9649

Epoch 31/35

16/16 [==============================] - 0s 8ms/step - loss: 0.0741 - accuracy: 0.9727 - val_loss: 0.1136 - val_accuracy: 0.9649

Epoch 32/35

16/16 [==============================] - 0s 10ms/step - loss: 0.0757 - accuracy: 0.9805 - val_loss: 0.1099 - val_accuracy: 0.9649

Epoch 33/35

16/16 [==============================] - 0s 10ms/step - loss: 0.0963 - accuracy: 0.9629 - val_loss: 0.1073 - val_accuracy: 0.9649

Epoch 34/35

16/16 [==============================] - 0s 10ms/step - loss: 0.0833 - accuracy: 0.9668 - val_loss: 0.1029 - val_accuracy: 0.9649

Epoch 35/35

16/16 [==============================] - 0s 10ms/step - loss: 0.0719 - accuracy: 0.9766 - val_loss: 0.1052 - val_accuracy: 0.9649
