# Supplementary material for: Early detection and analysis of accurate breast cancer for improved diagnosis using deep supervised learning for enhanced patient outcomes
Source: PeerJ Comput Sci. 2025 Apr 24;11:e2784. doi: 10.7717/peerj-cs.2784 (PMC12190644; doi:10.7717/peerj-cs.2784)
Supplement: Supplemental Information 8 — The results of Principal Component Analysis (PCA), including the explained variance for each principal component, highlighting how much variance is captured by each component, aiding in the reduction of dataset dimensions while retaining critical information for model training and analysis. [file peerj-cs-11-2784-s008.docx]

**Supplementary Table 2:** PCA Results

| Component \| | Variance Explained |
| --- | --- |
| 1 | 40% |
| 2 | 25% |
| 3 | 15% |
| 4 | 10% |
| 5 | 5% |
| 6 | 3% |
| 7 | 2% |
| 8 | 1% |
| 9 | 0.5% |
| 10 | 0.5% |
